# Phthalates and Their Impacts on Human Health

**DOI:** 10.3390/healthcare9050603

**Published:** 2021-05-18

**Authors:** Yufei Wang, Haifeng Qian

**Affiliations:** 1 College of Environment, Zhejiang University of Technology, Hangzhou 310032, China; jdwang@zjut.edu.cn; 2 School of Public Health and Preventive Medicine, Monash University, Melbourne 3000, Australia

**Keywords:** risk assessment, endocrine disruptors, plastics, health impact, child growth

## Abstract

Phthalates are a series of widely used chemicals that demonstrate to be endocrine disruptors and are detrimental to human health. Phthalates can be found in most products that have contact with plastics during producing, packaging, or delivering. Despite the short half-lives in tissues, chronic exposure to phthalates will adversely influence the endocrine system and functioning of multiple organs, which has negative long-term impacts on the success of pregnancy, child growth and development, and reproductive systems in both young children and adolescents. Several countries have established restrictions and regulations on some types of phthalates; however, we think that more countries should establish constraints or substitute measures for phthalates to reduce health risks. This article aims to summarize the adverse impacts of phthalates on human health, analyze the toxicity mechanism, assess the risks, and finally provide feasible strategies to reduce exposure of the public to phthalates.

## 1. Introduction

Plastic has brought great benefits to society since it was invented in 1907, however, it also has many negative impacts on the environment and human health, which has become a global problem. People are constantly exposed to plastics via contaminated food, packaging leachate (e.g., water bottle and medical devices), atmospheric fallout and urban dust containing microplastics, personal care products (PCPs) (e.g., cosmetic packaging), and synthetic clothing [1,2]. Long-term plastic exposure would inevitably lead to the leaching of many harmful substances. The most concerns include phthalates, bisphenol A (BPA), and polychlorinated biphenyls (PCB). These substances have been identified as endocrine-disrupting chemicals (EDCs) which interfere with normal hormonal actions [3,4]. Phthalates are a series of chemical substances, which are mainly used as plasticizers added to polyvinyl chloride (PVC) plastics for softening effects. Phthalates can potentially disrupt the endocrine system [5]. Health concerns regarding the detrimental impacts of phthalates on the development and reproductive system have been raised in the recent decades [6]. Compared to adults, children are much more vulnerable and sensitive to phthalates exposure, especially during early growth [7]. This review aims to summarize the impacts of phthalates on human health, especially on children, the mechanism and risk assessment of phthalates, and provide feasible strategies to reduce exposure of the public to phthalates.

## 2. Phthalates Applications and Exposure Routes

Phthalates, such as diethylhexyl phthalate (DEHP), dibutyl phthalate (DBP), diethyl phthalate (DEP), di-isononyl phthalate (DiNP), and di-iso-decyl phthalate (DiDP), are mainly used in the plastic industries as plasticizers to produce polyvinyl chloride (PVC). The short-branched low molecular weight phthalates, such as dimethyl phthalate (DMP) and DEP, are also widely produced and used in many industries, such as PCPs (e.g., hair products), pharmaceuticals, and medical devices (e.g., medical tubing) (Table 1). The global consumption of DEHP was estimated at 3.07 million tons (Global demand for plasticizers continues to rise, 2017). The estimated global market of phthalates in 2020 is expected to reach 10 billion USD and would still be widely used in plasticizers [8].

Plastic waste has received scrutiny by governmental and regulatory bodies. Global plastic use consumes more than 3 million tons of phthalates per year [9]. Due to their ubiquity in the environment, human exposure to phthalates leached from waste plastics is virtually unavoidable. For instance, in China, plastic usage tripled over eight years from 2003 and 2011, and reached over 50 million tons of raw plastics produced and estimated to keep increasing in the following years [9]. As a result, the relative higher exposure to phthalates was found in China due to the high usage of plastics. In the USA, more than 340 million pounds of phthalates are consumed every year and cause potential health and environmental risks [10]. Phthalates can be easily leaching into food, water, and other products applied directly to the human body. The detrimental health and environmental effects have been increasingly studied to assess the extent of the impacts on society. An important phthalate exposure route could be consisted of ingestion, inhalation, and dermal contact mainly via PCPs [9]. Some dairy products, fish, seafood, and oils are found to have a high level of phthalates. For the residents who live near phthalates manufacturing industries, phthalates are more likely to enter the body through absorption via the skin and the polluted air due to fugitive emission [10]. Phthalates are semi-volatile organic compounds (SVOCs). DEHP and DBP are the main compounds in both indoor and outdoor air phthalates [11]. Dermal absorption also occurs from the daily use of PCPs containing phthalates via plastic package. Infants are exposed to phthalates by drinking breast milk with their mothers exposed to DEHP and DiNP, and sucking on toys containing DEHP, DBP, and BBP [10]. Phthalates are also found to cross the placenta-blood barrier, which is the major exposure route of the fetus [12].

## 3. Bio-Metabolism of Phthalates in Human Body

**Figure 1:**
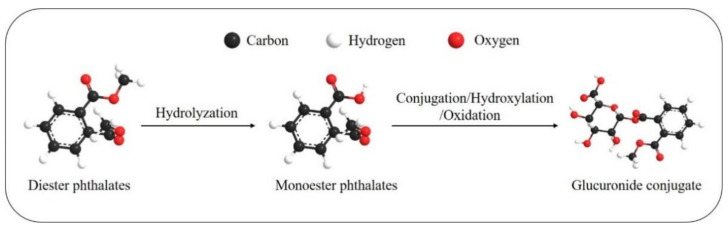
The metabolic pathway for phthalates.

The bio-metabolism in the human body is very rapid since phthalates have short biological half-lives, about 12 h [13]. Figure 1 presents the metabolic patterns. The first step of metabolism is hydrolyzation after absorption into cells. The second step is conjugation to form the hydrophilic glucuronide conjugate, which is catalyzed by the enzyme uridine 5′-diphosphoglucuronyl transferase [14]. The type of phthalates determines its toxicological fate in the body. Short-branched phthalates are often hydrolyzed to monoester phthalates and then excreted in the urine, while long-branched phthalates mainly undergo several bio-transformations, such as hydroxylation and oxidation, and then excreted in urine and feces as phase 2 conjugated compounds [15]. For example, the DEHP, which has complex branched chains, may be hydrolyzed to mono(2-ethylhexyl) phthalate (MEHP), mono(2-ethyl-5-hydroxyhexyl) phthalate, mono(2-ethyl-5-oxohexyl) phthalate, mono(2-ethyl-5-carboxypentyl) phthalate (MECPP), mono(2-carboxymethylhexyl) phthalate (MCMHP) or other metabolites. The metabolites of DEHP above could also be found in serum. According to the animal experiments, exposure to MEHP causes reproductive dysfunction in female zebrafishes, which is possibly due to the alteration in endocrine activities (elevated cortisol levels) [16]. In addition, according to half-life and distribution pattern, previous studies indicated that MECPP in urine and MCMHP in serum could be used as suitable biomarkers [14]. Most of the phthalates and their metabolites can be found in urine and feces, but some phthalates compounds (e.g., DEHP) and their metabolites can also be excreted in sweat [17]. Wittassek and Angerer found that the oxidative metabolism of DEHP is age-related. Younger children at the age of 6–7 years excrete more oxidative DEHP metabolites compared to mono-(2-ethylhexyl) phthalate (MEHP), one of the metabolites, than adults aged between 19 and 90 years [18].

## 4. Phthalates Toxicology and Risk Assessment

In rodent studies, it was found that phthalates have low acute toxicity with a median lethal dose (LD_50_) of 1–30 g/kg bodyweight, and its toxicity is mainly concentrated in the liver, kidney, thyroid gland tissue, and testis [6]. Evidence for adverse effects on reproduction and development in animals and humans is ample. According to the laboratory experiment on pregnant animals, exposure to DBP at 100 mg/kg bodyweight/day is toxic to fetal development [6]. The no-observed-adverse-effect level for DEHP to humans is 4.8 mg/kg bodyweight/day and the tolerate daily intake (TDI) is 48 μg/kg bodyweight [19]. Studies found that low molecular phthalates, such as DEP, can acutely irritate the skin, conjunctiva, and mucous membrane of the oral and nasal cavities [20]. Phthalate exposure is associated with adverse developmental effects in terms of increased prenatal mortality, reduced growth and birth weight, skeletal, visceral, and external malformations in rodents [6]. Experiments on male rats found that the nervous system is rather sensitive to low doses of DEHP exposure during puberty [21]. The impacts of phthalates on human beings vary from gene expression to physiological changes. High molecular weight phthalates exposure is found to cause methylation status of imprinted genes, which could be directly related to androgen response, estrogen response, protein secretion, and spermatogenesis [22,23]. Human epidemiological studies have shown a significant association between phthalates exposures and adverse reproductive outcomes in both women and men, for instance, type II diabetes and insulin resistance, overweight/obesity, allergy, asthma [24]. Among all phthalates, DEHP was most frequently tested and had the highest concentration in food, except in beef where di-n-octyl phthalate (DnOP) has the highest concentration [25]. In household dust, DEHP (median contamination level in indoor air: 400–700 ng/m^3^, (max. 410,000) mg/kg) has been found at high concentrations [6]. Evidence found that DEHP was significantly related to insulin resistance and higher systolic blood pressure and the reproduction system problems, including earlier menopause, low birth weight, pregnancy loss, and preterm birth [4]. During 2003–2004, the National Health and Nutrition Examination Survey (NHANES) found that the US population has been widely exposed to phthalates [26]. Women were found to be exposed at higher levels than men due to frequent use of PCPs (e.g., soaps and cosmetics) [26]. A systematic review and meta-analysis concluded that phthalates metabolites MBzP and MiBP were negatively associated with breast cancer among females [27]. Risk assessment of chemicals involves a comparison of the actual level of exposure to the acceptable level of exposure, mostly TDI values. But phthalates are a group of chemicals with individually different TDIs but with similar metabolites and impacts on the human body. Hence, the cumulative risk assessment is more appropriate to measure the risk of phthalates presented by summing the hazard quotient (HQ) as a hazard index (HI). Søeborg, et al. measuring the HQs and HIs of five phthalates, including DEHP, DBP, BBP, DINP, and DIDPA, found that DEHP and DBP contributed the greatest proportion of the HI. According to the NHANES data, the HI values of 10% of pregnant women exceeded 1, which means 10% of pregnant women were negatively impacted by phthalates, meanwhile, the Study for Future Families (SFF) found that the HI values of 4–5% of infants exceeded 1 [28].

## 5. Impacts of Phthalates on Children

When it comes to the impacts on children, epidemiological studies about phthalates toxicity focused on pregnancy outcomes, genital development, semen quality, precocious puberty, thyroid function, respiratory symptoms, and neurodevelopment [29]. Table 2 summarizes the health impacts on children. Among the epidemiological studies, it was revealed that exposure to phthalates adversely affected the level of reproductive hormones (luteinizing hormone, free testosterone, sex hormone-binding globulin), anogenital distance, and thyroid function [29]. Altered thyroid function is found to be associated with thyroid cancer [30]. A recent Chinese study concluded that phthalates exposure is related to the disrupted arginine and proline metabolism, resulting in the development of overweight and obesity among school-age children [31]. A 20-year birth cohort study found that prenatal phthalates exposure is negatively associated with height and weight during infancy and positively associated with height during childhood [32]. Another prospective study demonstrated that DiDP is associated with respiratory system health among boys aged under 5 years [33]. Phthalates have also been found to be linked to social impairment of children, the same as BPA [8]. Previous studies have found that infants and toddlers when contacting polymer toys may be exposed to levels of 5 to 44 μg/kg bodyweight/day of DiNP [6]. Later studies reported that around 20% of the children have been exposed to higher levels of phthalates than the cumulative TDI for DEHP and DBP [18]. In 2013–2014, over half of tests for phthalates for persons aged over 6 years found positive results for DEHP, and almost all women and children had DBP metabolites, according to the National Center for Health Statistics (NCHS) [10]. In Austria, few exceedances of TDI values of phthalates were observed among children, whereas the exceedances of TDI-based HIs for adults were in rare cases [34]. A study measuring the phthalates in air and dust in California (USA), found that 82–89% of children had DBP exposure exceeding the reproductive health benchmarks, and 8–11% of children aged less than 2 years exposed to DEHP exceeding cancer benchmarks [35]. A study conducted in China found that the cumulative risk because exposure to phthalates was higher in preschool children aged 3–6 years compared to the reports in German and Danish [36,37]. Rice, vegetables, and flour are the main sources of DEHP in China [38]. Xu, et al. reported that phthalates, mainly DEHP, DnBP, and DiBP, exist in commonly used plastic express packing bags, suggesting these bags may be the current main source of exposure of the population to phthalates [39]. In addition, the intake of vegetables grown in plastic greenhouses made children experience higher (nearly 3 times) DEHP and DnBP exposure than adults [40]. Foods containing fat (e.g., dairy and meat) tend to be more likely to absorb phthalates from the packaging. From the review of the literature, we believe that the exposure pathway depends on the food, air, or products containing phthalates.

## 6. Restrictions on Phthalates

Since the turn of the century, restrictions on phthalates have been proposed in many Asian and western countries (Table 3). In 2001, Japan prohibited DiNP and DEHP in toys and DEHP in food-handling gloves [41]. Since 2007, Europe banned DEHP, DBP, and BBP in all PVC and other plasticized materials in all toys and childcare articles, and DiNP, DiDP, and di-n-octyl phthalate (DnOP) for those products that can be placed in children’s mouth [42]. Recently, di-isobutyl phthalate (DiBP) was added to the restrictions in 2018 in 28 EU countries [43]. In 2008, the US Congress announced the Consumer Protection Safety Act (CPSA) that permanently banned the products, especially children’s toys and childcare articles, containing DEHP, DBP, and BBP at levels >0.1% by weight [44]. Australia also banned certain products that contained more than 1% of DEHP and could be chewed or sucked by children up to and including 36 months of age [45]. This ban also applied to food vessels and utensils, besides toys and childcare articles. Similar restrictions also have been announced for the exported products from China, the biggest manufacturer and consumer of phthalates, and Canada. The latest phthalates regulations in China in 2017 set detection limitations of 16 phthalates in food, food containers, and packing materials (GB 5009.271-2016, GB/T 21928-2008, GB 9685-2016, GB 15593-1995). Dissolved DEHP detected in transfusion (infusion) equipment should not be more than 10 mg/mL (GB 14232.1-2004/ISO 3826-1, GB 24613-2009). It has been reported that over 100 healthcare institutions around the world are reducing the use of PVC and phthalates [46].

## 7. Strategic Recommendations

Current evidence and research suggest the need for further restrictions and strategies to reduce the exposure to phthalates. Feasible strategies are as following:

(1) Reassess and apply the restrictions where appropriate on high-risk products. DEHP, DBP, BBP, DiNP, DiDP, DnOP, and DiBP are now limited in certain products in many countries, which could contribute to reducing phthalates exposure. Less exposure will be observed if similar restrictions are applied in those countries that do not currently apply phthalates restrictions. Products with high phthalates exposure risk need to be strictly limited, such as food packaging, PCPs, medical devices, products likely to be sucked or ingested by infants, children, and adolescents. Pregnant women and lactating women have also been identified as vulnerable groups, and it is, therefore, necessary to impose stricter standards on the products that contact such specific groups of people.
(2) Phthalate alternatives (PA) with less toxicity and leakage should also be considered, especially in the health care industry. The leaching of DEHP in the environment is uncontrollable but can be avoided by using DEHP-free alternatives [47]. The concentration of phthalates needs to be limited in the products that are most frequently used but people are not aware of the toxic exposure. In the medical industry, trioctyl trimellitate (TOTM) and diisononylester (DINCH) are found to be promising DEHP alternatives (Thomas, et al., 2021). Besides, epoxidised soybean oil (ESBO), di-(2-ethylhexyl) terephthalate (DEHT), and acetyl tributyl citrate (ATBC) are popular PAs in the plasticizer market. In Europe, PVC-free medical and DEHP-free devices are available, by using polyethylene, polypropylene, polyurethane, and other polyolefins, silicone, ethylene-vinyl acetate, and multi-layerlaminate plastics as alternatives [47]. The alternative softeners are citrates, benzoates, trimellitates, and adipates, with much lower toxic levels than DEHP (Ruzickova, et al., 2004). Where practicable, using glass containers instead of plastic packaging, avoiding heating food in plastic containers, avoiding using fragrance that may contain phthalates, and reading the label of PCPs can easily reduce the exposure to phthalates in daily life [47]. Also, testing drinking water routinely for phthalates can keep residents at safe levels of exposure. When processing food, it is advisable to use phthalate-free gloves, utensils, and packaging to reduce exposure in food. The most recent exposure assessment found decreased phthalates and increased PA in humans [48]. Few adolescent participants were detected to have higher estimated daily intakes than health-based guidance values.
(3) To protect children, soft vinyl toys, old plastic toys, and teething rings should be avoided. Children should be kept away from waste sites of factories, especially plastic manufacturers, which can help to avoid dermal and airborne intake. A recent study at a kindergarten in China found that there were relatively high phthalates exposures in indoor air and dust, compared to the outdoor environment [49]. In the community, it is also necessary to limit and measure the level of phthalates in kindergarten, schools, hospitals, and shopping malls. Even though PA use may reduce the phthalates levels, PA exposure cannot be ignored as well, especially from tap water and air particles [50]. Public awareness needs to be improved to educate vulnerable members of the community to avoid using plastics voluntarily, especially plastics containing phthalates.

## 8. Conclusions

**Figure 2:**
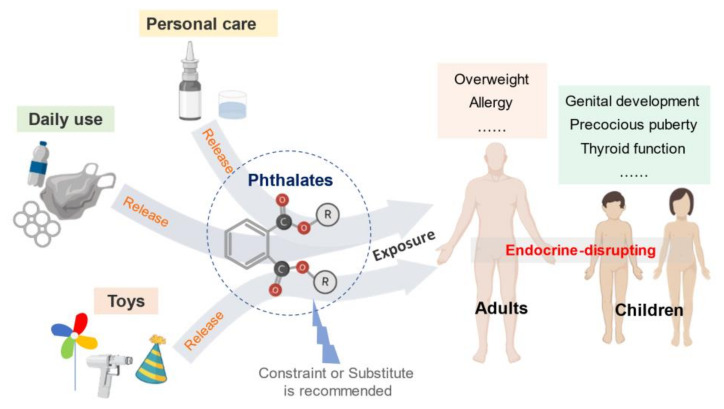
Phthalates application and the impacts on human health.

While the benefits of plastics are enjoyed worldwide, the environment and human health are adversely influenced (Figure 2). Phthalates, as endocrine-disrupting chemicals and SVOCs, are detrimental to the reproductive, neurological, and developmental systems of human from multiple exposure pathways. Children are at a higher level of exposure and more vulnerable to phthalates. Currently, many phthalates are banned and restricted in multiple countries. Plastic manufacturers and suppliers are required to understand plastic regulations to meet national and international standards. Till now, information related to occupational exposure to phthalates remains limited. Further research is required to assess the risk of occupational exposure to phthalates.

## Figures and Tables

**Table 1:** Common phthalate compounds.

Compounds	Abbreviation	Where It Can Be Found	Also Named As
Diethylhexyl phthalate	DEHP	Plasticizer	Di-2-ethylhexyl phthalate; Bis-2-ethylhexyl phthalate.
Dibutyl phthalate	DBP	Nail polishers; plasticizer; an additive to adhesives or printing inks;	Di-n-butyl phthalate, DnBP, DNBP
Diethyl phthalate	DEP	Toothbrushes; automobile parts; tools; toys; food packaging; cosmetics; insecticides; aspirin	Ethyl phthalate; Di-n-ethyl phthalate
Di-isononyl phthalate	DiNP	Plasticizer	Bis(7-methyloctyl) phthalate; DINP
Di-iso-decyl phthalate	DiDP	Plasticizer	Di(i-decyl) phthalate; diisodecyl phthalate; DIDP
Butyl benzyl phthalate	BBP	Plasticizer	Benzyl n-butyl phthalate; n-Butyl benzyl phthalate
Mono-(2- ethylhexyl) phthalate	MEHP	Vinyl tiles; food conveyor belts; carpet tile; artificial leather	tert-Butyldimethylsilyl 2-ethylhexyl phthalate
Di-isobutyl phthalate	DiBP	Plasticizer; adhesive	Di(i-butyl)phthalate; Isobutyl phthalate; di-l-butyl phthalate; DIBP
Dioctyl phthalate	DnOP	Household items and building products; food applications	Di-n-octyl phthalate; DNOP

**Table 2:** Health impacts on children.

Category	Health Concerns
Endocrine systems	Weight (overweight and obesity) and height
	Type II diabetes and insulin resistance
	Thyroid function and increased risk of thyroid cancer
	Higher systolic blood pressure
	Anogenital distance
	Precocious puberty
	Males: genital development, semen quality
	Females: pregnancy outcome (pregnancy loss and preterm birth, low birth weight), reproductive hormones (including lueinizing hormone, sex hormone-binding globulin, earlier menopause)
Others	Respiratory system: allergy and asthma Nervous system: delayed neurodevelopment, social impairment

**Table 3:** Restrictions in Japan, Europe, the US, Australia, and China.

Country	Restrictions
Japan [41]	DiNP and DEHP are banned in toys; DEHP is banned in food-handling gloves
Europe [42,43]	DEHP, DBP, DiBP, and BBP are banned in all PVC and plasticized toys and childcare articles; DiNP, DiDP, and DnOP are banned for products that can be placed in children’s mouth
The United States [44]	Products containing DEHP, DBP, and BBP at levels >0.1% by weight shall be banned, especially children’s toys, and childcare articles; children’s products that can be placed in a child’s mouth or childcare articles containing more than 0.1% of DiNP, DiDP, and DnOP are banned
Australia [45]	Children’s plastic products containing, or have a component containing more than 1% by weight DEHP are banned
China (National Standard of the People’s Republic of China)	16 phthalates are restricted in food and food containers, including DNP, DnOP, DEHP, DiNP, DiBP, BBP, etc; dissolved DEHP in transfusion (infusion) equipment is restricted to less than 10 mg/mL; the total amount of DEHP, BBP, DBP in childcare articles should not be more than 0.1%.

## References

[1] Cook C.R., Halden R.U. (2020). Ecological and health issues of plastic waste. Plastic Waste and Recycling.

[2] Ke M.J., Ye Y.Z., Zhang Z.Y., Gillings M., Qu Q., Xu N.H., Xu L.S., Wang J.D., Qian H.F. (2021). Synergistic effects of glyphosate and multiwall carbon nanotubes on Arabidopsis thaliana physiology and metabolism. Sci. Total Environ..

[3] Schug T.T., Johnson A.F., Birnbaum L.S., Colborn T., Guillette L.J., Crews D.P., Collins T., Soto A.M., Vom Saal F.S., McLachlan J.A. (2016). Minireview: Endocrine disruptors: Past lessons and future directions. Mol. Endocrinol..

[4] Grindler N.M., Vanderlinden L., Karthikraj R., Kannan K., Teal S., Polotsky A.J., Powell T.L., Yang I.V., Jansson T. (2018). Exposure to phthalate, an endocrine disrupting chemical, alters the first trimester placental methylome and transcriptome in women. Sci. Rep..

[5] Verma R., Vinoda K.S., Papireddy M., Gowda A.N.S. (2016). Toxic pollutants from plastic waste-a review. Procedia Environ. Sci..

[6] Heudorf U., Mersch-Sundermann V., Angerer J. (2007). Phthalates: Toxicology and exposure. Int. J. Hyg. Environ. Health.

[7] Chou Y.Y., Huang P.C., Lee C.C., Wu M.H., Lin S.J. (2009). Phthalate exposure in girls during early puberty. J. Pediatric Endocrinol..

[8] Benjamin S., Masai E., Kamimura N., Takahashi K., Anderson R.C., Faisal P.A. (2017). Phthalates impact human health: Epidemiological evidences and plausible mechanism of action. J. Hazard. Mater..

[9] Wang W., Leung A.O.W., Chu L.H., Wong M.H. (2018). Phthalates contamination in China: Status, trends and human exposure-with an emphasis on oral intake. Environ. Pollut..

[10] America’s Children and the Environment, 3rd ed.; Phthalates; 2017. https://www.epa.gov/sites/production/files/2017-08/documents/phthalates_updates_live_file_508_0.pdf.

[11] Jia S., Sankaran G., Wang B., Shang H., Tan S.T., Yap H.M., Shen J., Gutiérrez R.A., Fang W., Liu M. (2019). Exposure and risk assessment of volatile organic compounds and airborne phthalates in Singapore’s Child Care Centers. Chemosphere.

[12] Dutta S., Haggerty D.K., Rappolee D.A., Ruden D.M. (2020). Phthalate exposure and long-term epigenomic consequences: A review. Front. Genet..

[13] Hoppin J.A., Brock J.W., Davis B.J., Baird D.D. (2002). Reproducibility of urinary phthalate metabolites in first morning urine samples. Environ. Health Perspect..

[14] Frederiksen H., Skakkebaek N.E., Andersson A.M. (2007). Metabolism of phthalates in humans. Mol. Nutr. Food Res..

[15] Koch H.M., Bolt H.M., Preuss R., Angerer J. (2005). New metabolites of di (2-ethylhexyl) phthalate (DEHP) in human urine and serum after single oral doses of deuterium-labelled DEHP. Arch. Toxicol..

[16] Park C.B., Kim G.E., Kim Y.J., On J., Park C.G., Kwon Y.S., Pyo H., Yeom D.H., Cho S.H. (2020). Reproductive dysfunction linked to alteration of endocrine activities in zebrafish exposed to mono-(2-ethylhexyl) phthalate (MEHP). Environ. Pollut..

[17] Genuis S.J., Beesoon S., Lobo R.A., Birkholz D. (2012). Human elimination of phthalate compounds: Blood, urine, and sweat (BUS) study. Sci. World J..

[18] Wittassek M., Angerer J. (2008). Phthalates: Metabolism and exposure. Int. J. Androl..

[19] Lyche J.L., Gutleb A.C., Bergman Å., Eriksen G.S., Murk A.J., Ropstad E., Saunders M., Skaare J.U. (2009). Reproductive and developmental toxicity of phthalates. J. Toxicol. Environ. Health Part B.

[20] Mikula P., Svobodova Z., Smutna M. (2005). Phthalates: Toxicology and food safety-a review. Czech J. Food Sci..

[21] Capela D., Mhaouty-Kodja S. (2021). Effects of pubertal exposure to low doses of di-(2-ethylexyl) phthalate on reproductive behaviors in male mice. Chemosphere.

[22] Chen C.H., Jiang S.S., Chang I.S., Wen H.J., Sun C.W., Wang S.L. (2018). Association between fetal exposure to phthalate endocrine disruptor and genome-wide DNA methylation at birth. Environ. Res..

[23] Tindula G., Murphy S.K., Grenier C., Huang Z., Huen K., Escudero-Fung M., Bradman A., Eskenazi B., Hoyo C., Holland N. (2018). DNA methylation of imprinted genes in Mexican–American newborn children with prenatal phthalate exposure. Epigenomics.

[24] Wang Y., Zhu H., Kannan K. (2019). A review of biomonitoring of phthalate exposures. Toxics.

[25] Schecter A., Lorber M., Guo Y., Wu Q., Yun S.H., Kannan K., Hommel M., Imran N., Hynan L.S., Cheng D. (2013). Phthalate concentrations and dietary exposure from food purchased in New York State. Environ. Health Perspect..

[26] Center of Disease Control and Prevention (CDC) Phthalates Factsheet. National Biomonitoring Program. April 2017. https://www.cdc.gov/biomonitoring/Phthalates_FactSheet.html.

[27] Liu G., Cai W., Liu H., Jiang H., Bi Y., Wang H. (2021). The Association of Bisphenol A and Phthalates with Risk of Breast Cancer: A Meta-Analysis. Int. J. Environ. Res. Public Health.

[28] Søeborg T., Frederiksen H., Andersson A.M. (2012). Cumulative risk assessment of phthalate exposure of Danish children and adolescents using the hazard index approach. Int. J. Androl..

[29] Jurewicz J., Hanke W. (2011). Exposure to phthalates: Reproductive outcome and children health. A review of epidemiological studies. Int. J. Occup. Med. Environ. Health.

[30] Alsen M., Sinclair C., Cooke P., Ziadkhanpour K., Genden E., van Gerwen M. (2021). Endocrine Disrupting Chemicals and Thyroid Cancer: An Overview. Toxics.

[31] Xia B., Zhu Q., Zhao Y., Ge W., Zhao Y., Song Q., Zhou Y., Shi H., Zhang Y. (2018). Phthalate exposure and childhood overweight and obesity: Urinary metabolomic evidence. Environ. Int..

[32] Berman Y.E., Doherty D.A., Main K.M., Frederiksen H., Hickey M., Keelan J.A., Newnham J.P., Hart R.J. (2021). Associations between Prenatal Exposure to Phthalates and Timing of Menarche and Growth and Adiposity into Adulthood: A Twenty-Years Birth Cohort Study. Int. J. Environ. Res. Public Health.

[33] Vernet C., Pin I., Giorgis-Allemand L., Philippat C., Benmerad M., Quentin J., Calafat A.M., Ye X., Annesi-Maesano I., Siroux V. (2017). In utero exposure to select phenols and phthalates and respiratory health in five-year-old boys: A prospective study. Environ. Health Perspect..

[34] Sui H., Jiang D., Wu P., Zhang L., Liu Z., Yang D. (2015). Dietary intake and risk assessment of diethylhexyl phthalate in Chinese populations. Zhonghua Yu Fang Yi Xue Za Zhi Chin. J. Prev. Med..

[35] Lioy P.J., Hauser R., Gennings C., Koch H.M., Mirkes P.E., Schwetz B.A., Kortenkamp A. (2015). Assessment of phthalates/phthalate alternatives in children’s toys and childcare articles: Review of the report including conclusions and recommendation of the Chronic Hazard Advisory Panel of the Consumer Product Safety Commission. J. Expo. Sci. Environ. Epidemiol..

[36] Gaspar F.W., Castorina R., Maddalena R.L., Nishioka M.G., McKone T.E., Bradman A. (2014). Phthalate exposure and risk assessment in California child care facilities. Environ. Sci. Technol..

[37] Koch H.M., Wittassek M., Brüning T., Angerer J., Heudorf U. (2011). Exposure to phthalates in 5–6 years old primary school starters in Germany—A human biomonitoring study and a cumulative risk assessment. Int. J. Hyg. Environ. Health.

[38] Gao H., Huang K., Wu X.Y., Cai X.X., Han Y., Zhu P., Hao J.H., Tao F.B. (2019). Cumulative risk assessment of phthalates exposure in preschool children. Zhonghua Liu Xing Bing Xue Za Zhi.

[39] Xu Z., Xiong X., Zhao Y., Xiang W., Wu C. (2020). Pollutants delivered every day: Phthalates in plastic express packaging bags and their leaching potential. J. Hazard. Mater..

[40] Zhang Y., Huang B., Thomsen M., Sabel C.E., Hess F., Hu W., Tian K. (2018). One overlooked source of phthalate exposure-oral intake from vegetables produced in plastic greenhouses in China. Sci. Total Environ..

[41] Mutsuga M., Wakui C., Kawamura Y., Maitani T. (2002). Isolation and identification of some unknown substances in disposable nitrile-butadiene rubber gloves used for food handling. Food Addit. Contam..

[42] EUR-Lex EU Phthalates Directive 2005/84/EC. 14 December 2005. https://eur-lex.europa.eu/legal-content/EN/TXT/?uri=CELEX:32005L0084.

[43] Commission Regulation (EU) 2018/2005 Official Journal of the European Union. 17 December 2018. https://eur-lex.europa.eu/legal-content/EN/TXT/PDF/?uri=CELEX:32018R2005&from=EN.

[44] Consumer Product Safety Improvement Act of 2008 (CPSIA). Public Law 110–314. 14 August 2008. https://www.congress.gov/110/plaws/publ314/PLAW-110publ314.pdf.

[45] Product Safety Australia DEHP in Children’s Plastic Items. Australian Competition & Consumer Commission. n.d. https://www.productsafety.gov.au/bans/dehp-in-childrens-plastic-items.

[46] Center of Health, Environment & Justice PVC Policies across the World. n.d. http://www.chej.org/pvcfactsheets/PVC_Policies_Around_The_World.html.

[47] Hartmann C., Uhl M., Weiss S., Koch H.M., Scharf S., König J. (2015). Human biomonitoring of phthalate exposure in Austrian children and adults and cumulative risk assessment. Int. J. Hyg. Environ. Health.

[48] Bastiaensen M., Gys C., Colles A., Malarvannan G., Verheyen V., Koppen G., Govarts E., Bruckers L., Morrens B., Franken C. (2021). Biomarkers of phthalates and alternative plasticizers in the Flemish Environment and Health Study (FLEHS IV): Time trends and exposure assessment. Environ. Pollut..

[49] Wang L., Wu Z., Gong M., Xu Y., Zhang Y. (2020). Non-dietary exposure to phthalates for pre-school children in kindergarten in Beijing, China. Build. Environ..

[50] Li N., Ying G.G., Hong H., Tsang E.P.K., Deng W.J. (2021). Plasticizer contamination in the urine and hair of preschool children, airborne particles in kindergartens, and drinking water in Hong Kong. Environ. Pollut..

